# Evaluation and Comparison of Five-Year Survival of Tooth-Supported Porcelain Fused to Metal and All-Ceramic Multiple Unit Fixed Prostheses: A Systematic Review

**DOI:** 10.7759/cureus.30338

**Published:** 2022-10-15

**Authors:** Prabha Shakya Newaskar, Subhash Sonkesriya, Rashmi Singh, Umesh Palekar, Hiroj Bagde, Ashwini Dhopte

**Affiliations:** 1 Department of Prosthodontics, Rural Dental College, Pravara Institute of Medical Sciences - Deemed University (PIMS-DU), Loni, IND; 2 Department of Prosthodontics, Government Dental College and Hospital, Indore, IND; 3 Department of Prosthodontics, Mansarovar Dental College, Hospital and Research Centre, Bhopal, IND; 4 Periodontology, Rama Dental College and Research Centre, Kanpur, IND; 5 Oral Medicine and Radiology, Rama Dental College and Research Centre, Kanpur, IND

**Keywords:** survival rate, success rate, metal free prosthesis, metal ceramic, mechanical failure, biological failure

## Abstract

The prosthesis must have good survival despite being functional for at least 5-10 years. This makes sure that the replacement of missing teeth does not become a repeated expense. Of 579 identified articles, 15 met the inclusion criteria for systematic review. Missing teeth replacement materials are divided into two groups: porcelain fused to metal and all ceramics. Data related to survival rates as well as the most common mode of failure is observed from both groups. It was observed that porcelain fused to metal prostheses had an approximately 99.5% survival rate and an approximately 92% survival rate for all-ceramic tooth-supported prostheses after five years of insertion. Porcelain-fused-to-metal (PFM) prostheses had a better survival rate after five years of insertion as compared to all-ceramic prostheses. Porcelain fused to metal should be the treatment of choice for dentists and patients when missing teeth need to be fixed.

## Introduction and background

Fixed prostheses are used to replace lost teeth in the mouth and are supported by natural teeth. The teeth on either side of the edentulous area are employed to support the prosthesis in this case. There are two types of materials used for its fabrication: porcelain-fused-to-metal (PFM) and all-ceramic. In the case of PFM restoration, there is a porcelain veneer supported by a metal framework. While in all-ceramic restorations, both the framework and the veneer layer are made of ceramic. PFM prostheses have been used successfully for decades. However, with the focus of patients shifting toward aesthetics, all-ceramic prostheses are increasingly in demand [1]. The strength and durability of the prosthesis is the main reason why PFM restorations work so well [2,3]. Studies demonstrating that PFM prostheses are superior in strength but inferior in aesthetics to all-ceramic preparations are scarce [4,5]. According to Anusavice [6], "Restoration success is defined as the demonstrated ability of a restoration (including a prosthesis) to perform as expected." Pjetursson et al. [7] defined success as a fixed partial denture (FPD) remaining unmodified and free of problems for the whole monitoring period. Clinical indices such as United States Public Health Service (USPHS)/Ryge criteria [8], CDA criteria [9], and Hickel's criteria [10] have been created to standardise the restoration evaluation criteria.

A restoration failure is any problem that necessitates prosthesis replacement. Conditions that constitute restoration failure include secondary caries, excessive wear of the opposing tooth surface, irreversible pulpitis, excessive erosion and roughening of the ceramic surface, unacceptable esthetics, ditching of the cement margin, cracking, chipping, and bulk fracture [6]. Despite their great success as restorations, PFMs usually face marginal defects as one of the most common failures. Due to this, the aesthetics are compromised, and therefore the prosthesis would need to be replaced. Chipping is another complication endured, but it requires no more than veneering and polishing [11,12]. Substructure fracture is rarely seen but is a complication nonetheless. Despite the failure, it is a cost-effective treatment option for the average person, and its fabrication requires no special equipment. All ceramic preparations, moreover, are known to have chipping fractures. While fractures in the posterior region pose a functional problem, fractures in the anterior region raise aesthetic issues. In either case, mechanical failures in prostheses happen over some time, and they are usually multifactorial, which include factors of the material used for preparation, improper masticatory forces, and lab technical faults. Compared to PFM, it is more expensive and requires the use of special equipment to make.

Every day, clinicians face treatment difficulties for their patients. Patient preferences and clinician experience should be considered when making treatment decisions. Thus, practitioners must recognise high-quality data and only utilise it to support their everyday practice. The current study aims to investigate the survival rate and most common modes of failure endured by multi-unit prostheses fabricated with PFM and all-ceramics.

## Review

The focused PICO question was "Which material, out of porcelain-fused-to-metal and all-ceramic, has a higher success and survival rate for replacing lost teeth in partially edentulous patients after five years of use?" and the most common modes of failure were observed in both materials (Table 1).

**Table 1:** PICO guidelines for inclusion criteria PICO: population, intervention, comparison, and outcomes

Question	Inclusion
P	All patients aged over 20 years that have undergone treatment and received multi-unit fixed prosthesis
I	Prosthesis fabricated with porcelain fused to metal
C	Prosthesis fabricated with all ceramic
O	Survival and success rate of both prosthesis

This systematic review was developed using the Preferred Reporting Items for Systematic Reviews and Meta-Analyses (PRISMA) checklist [12]. The following electronic databases were searched: PubMed/MedLine, Cochrane, Scopus, EBSCO Host, Quintessence Publication, and Google Scholar using search terms (MeSH terms) survival rate AND modes of failure AND (porcelain-fused-to-metal OR metal ceramic) AND (all-ceramic OR zirconia OR monolithic) AND multiple-units AND anterior prosthesis AND posterior prosthesis AND (fixed prosthesis OR dental bridges OR Fixed Partial Dentures OR fixed dental prosthesis) retrospective studies, comparative studies, randomized clinical trials, and cohort studies. The searches were limited to the English language, humans, and clinical trials conducted from September 2012 up to October 2021.

**Figure 1:**
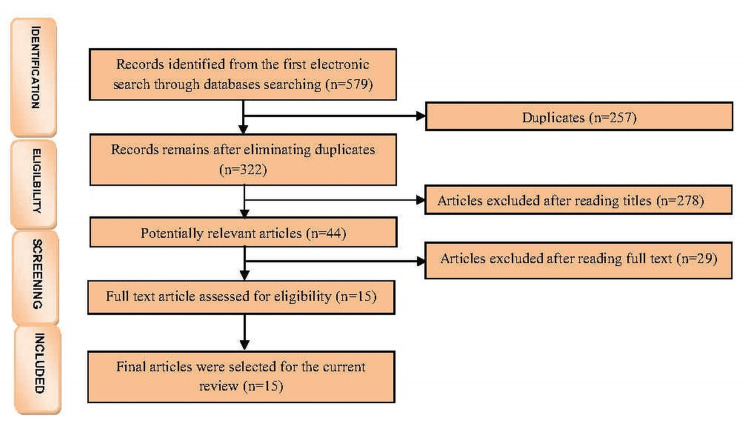
PRISMA flow diagram representing final number of articles selected. PRISMA: Preferred Reporting Items for Systematic Reviews and Meta-Analyses.

After the elimination of duplicate records, titles and abstracts were independently screened. The reviewers agreed upon the selection of 15 articles (Figure 1), warranting full-text access.

The following are inclusion criteria: retrospective observational studies and randomised controlled trials with a minimum sample size of 15 which compare the modes of failure and survival rates of porcelain fused to metal and all-ceramic tooth-supported fixed prostheses [13]. The records were tabulated, and the comparison parameters were percentage survival after five years, mean age of the patients, location of the prosthesis, number of units, type of material used, and most to least standard modes of failure (Table 2).

**Table 2:** Assessments studying the survival and failure modes of porcelain fused to metal prosthesis FPD: fixed partial denture

Author	Type of study	Participants	Material studied	Location	Percentage survival after five years	Modes of failure (number of cases or percent cases)	Observation/conclusion
Pelaez et al. [14]	Randomized controlled trial	37	Porcelain fused to metal and all-ceramic	Arch not mentioned, posterior	Metal-ceramic: 100% all-ceramic: 95%	2% ceramic chipping 4% marginal exposure no statistically significant difference seen in biological complications	Survival rates for metal-ceramic and zirconia restorations were 100% and 95%
Sorrentino et al. [15]	Prospective clinical trial	37	All-ceramic veneered ceramic	Arch not mentioned, posterior	100%	3% chipping 6% occlusal wear 3% marginal integrity 4% anatomical form	100% cumulative survival rate and 95.4% cumulative success rates
Perry et al. [16]	Prospective clinical study	15	All-ceramic veneered ceramic	Arch not mentioned, anterior and posterior	100%	3% chipping	93.75% of bridges are marginally integrated, and 93.75% have good periodontal health
Lops et al. [17]	Prospective clinical trial	28	All-ceramic veneered ceramic	Arch not mentioned, posterior	85%	16% chipping 3% loss of vitality 1% secondary caries 1% endodontic complications 2% periodontal pathology	Cumulative survival and success rates were 88.9% and 81.8%, respectively
Hey et al. [18]	Clinical trial	23	Metal-ceramic	Arch not mentioned, anterior and posterior	88%	10% porcelain chipping 1% substructure fracture 1% biologic failure	Success rate was calculated at 58.6% and the survival rate at 88%.
Rinke et al. [19]	Prospective study	75	All-ceramic veneered ceramic	Maxillary and mandibular posterior	75%	31% chipping 4% framework fracture 7% loss of retention 6% secondary caries 5% loss of vitality	Survival and success rates of zirconia-based posterior FPDs were inferior to those published for metal-ceramic FPDs
Burke et al. [20]	Randomized controlled trial	36	All-ceramic veneered ceramic	Maxillary anterior and maxillary and mandibular posterior	97%	7% chipping	97% survival rate
Chaar et al. [21]	Randomized controlled trial	58	All-ceramic veneered ceramic	Maxillary and mandibular posterior	93.6%	4% framework fractures, 2% secondary caries	In-Ceram Zirconia presented a 10-year survival rate (93.6%) similar to that reported for conventional FPDs
Naenni et al. [22]	Randomized controlled trial	40	All-ceramic veneered ceramic	Maxillary and mandibular posterior	100%	30% chipping,18% surface roughness	The survival rate was 100% for both test and control FPDs
Sola-Ruiz et al. [23]	Prospective study	27	All-ceramic veneered ceramic	Arch did not mention anterior	88.9%	5% chipping, 2% loss of retention 1% periapical pathology	The clinical success rate was 88.8% after the 7-year follow-up
Selz et al. [24]	Prospective study	24	All-ceramic veneered ceramic	Arch not specified, anterior and posterior	100%	2% chipping, 2% loss of retention, 1% colour instability, 14% surface roughness	Survival rate and success rate of the FPDs were 100% and 91.7%
Ioannidis and Bindl [25]	Prospective study	55	All-ceramic veneered ceramic	Maxillary and mandibular Posterior	85%	16% chipping, 3% loss of vitality, 1% secondary caries, 1% endodontic complications, 2% periodontal pathology	10-year cumulative survival rate amounted to 85.0%
Teichmann et al. [26]	Prospective study	17	All-ceramic veneered ceramic	Arch not mentioned, anterior and posterior	95%	8% chipping 1% periodontal pathology	10-year survival rate and 10-year chipping-free rates were 95.0% and 78.8%
Boening and Ullmann [27]	Retrospective study	18	Metal-ceramic	Mandibular anterior and maxillary and mandibular posterior	89%	In this study, the survival was checked in bruxism patients	The survival rate with the event "any restoration complication" dropped to 84% after 77 months and then remained constant
Kavaz et al. [28]	Randomized controlled trial	90	Porcelain fused to metal and metal-acrylic	Arch not mentioned, anterior and posterior	Metal-ceramic: 98% metal acrylic: 82%	48% ceramic chipping, 23% catastrophic fracture, 45% marginal exposure, 52% gingival swelling, 28% calculus formation	When the rate of complications increased, and the duration of using prostheses decreased

Exclusion criteria were in-vitro studies, ex-vivo studies, animal studies, case reports, review articles, protocols, clinical guidelines, and editorial letters; articles in other languages were excluded. After reading the full text, 29 studies were excluded due to exclusion criteria that are mentioned in Table 3.

**Table 3:** Excluded articles with reasons

References	Reason for exclusion
Anusavice [6]	Standardized the modes of failure, complications, and measurement of survival rate
Maló et al. [29]	The study considered implant-supported fixed prostheses
Schwarz et al. [30]	Observational study on implant supported prosthesis
Biscaro et al. [31]	In vivo study comparing ceramic and porcelain fused to metal single crowns
Reitemeier et al. [32]	Randomized controlled trial comparing metal ceramic single crowns and fixed dental prosthesis
Esquivel-Upshaw et al. [33]	Randomized controlled trials considered implant-supported fixed prostheses
Zafar and Ghani [34]	Cross-sectional study about immediate complications
Konstantinidis et al. [35]	Prospective evaluation of all-ceramic implant and tooth-supported restorations
Le et al. [36]	A systematic review on the clinical success of tooth- and implant-supported all-ceramic-based fixed dental prostheses
Pjetursson et al. [37]	A previous systematic review about multi-unit tooth-supported crown
Sailer et al. [38]	A systematic review of single crowns
Walton [39]	A cohort study comparing implant-supported and tooth-supported multi-unit prosthesis
Pang et al. [40]	Randomized control studying fracture mechanisms in retrieved prosthesis
Varol and Kulak-Özkan [41]	An in-vitro study comparing the fit of single crowns.
Karl [42]	A systematic review comparing resin-bonded, all-ceramic and Porcelain fused to metal FDPs
Abou-Ayash et al. [43]	A systematic review on implant-supported prosthesis
Heintze et al. [44]	In vitro Study on fatigue testing for porcelain fused to metal crowns
Vafaee et al. [45]	A systematic review on implant-supported prosthesis
Holm et al. [46]	A systematic review about implant-supported multi-unit fixed prosthesis
Pott et al. [47]	Compared all-ceramic single crowns and FPD
Lemos et al. [48]	A systematic review on comparing porcelain fused to metal and ceramic implant supported prosthesis
Papaspyridakos et al. [49]	Retrospective study about metal ceramic implant-supported prosthesis
Reitemeier et al. [50]	Prospective study on the clinical outcome of metal-ceramic crowns
Forrer et al. [51]	Cohort study comparing the survival of lithium di-silicate material with metal crowns, implant supported.
Hu et al. [52]	Previous systematic review comparing the complication rates of Implant supported prosthesis
Nejatidanesh et al. [53]	A retrospective study considered implant-supported fixed prostheses
Rammelsberg et al. [54]	Cohort study about implant-supported and combined tooth-implant-supported porcelain fused to metal and ceramic fixed dentures
Alsterstål-Englund et al. [55]	Retrospective evaluation of implant-supported restorations
Rauch et al. [56]	A survey conducted amongst German dentists regarding material selection for tooth-supported single crowns

Results

Of 579 identified articles, 15 met the inclusion criteria for systematic review (Figure 1). Missing teeth replacement materials are divided into two groups: porcelain fused to metal and all ceramics. Pelaez et al. [14] suggested a 100% survival rate for PFM in the posterior region, and Hey et al. [18] suggested an 88% survival rate for PFM in the anterior and posterior regions. Researchers [19-22,24-28] proposed an 88-95% survival rate for the all-ceramic posterior region, while Sola-Ruiz et al. [23] proposed an 89% survival rate for the all-ceramic anterior region.

Discussion

In the past, there have been very few systematic reviews comparing the survival rates of porcelain fused to metal and all-ceramic tooth-supported restorations. The present systematic review brings to light the literature collected in the last nine years. It becomes clear that while porcelain fused to metal is superior in strength, all-ceramic is superior in terms of aesthetics. Each material, therefore, fulfils a crucial purpose.

The duration of this review was selected from January 2012 because, at the same time, Anusavice [6] gave standardised criteria for the success, survival, and failure of any FPD prosthesis. From Table 1, it is clear that the most commonly seen failure in PFM tooth-supported prostheses was veneer chipping [16-19,21-26], which led to the exposure of the metallic substructure. Catastrophic fractures occur very rarely. The most frequently occurring biological failures were periodontal pocket formation and gingival swelling [25-26,28]. Secondary caries was seen in a few cases as well [17,19,21,25]. The survival of PFM prosthesis was seen to be the lowest (88%) when it was studied in patients with bruxism as a para-functional habit. The current review results were similar to previous studies. When compared to patients who did not have such habits, the five-year survival percentage ranged from 93% to 100%.

In all-ceramic prostheses, it has become evident that ceramic chipping is also the most common mode of failure. This kind of failure kept happening because the surface of the area was rough [24]. Failures like compromised marginal integrity and framework fractures [19,21] were also observed. The most repeated biological failures were endodontic complications [17,25-26] and periodontal pathology [19,25]. The lowest survival percentage was 75%, as seen in a study conducted by Rinke et al. [19]. The average range of the five-year survival percentage was 85-95% [14,17,20-21,23,25-26].

An approximate 99.5% survival rate for PFM tooth-supported prostheses and an approximately 92% survival rate for all-ceramic tooth-supported prostheses after five years of insertion were estimated. Our study results showed that, according to a systematic review conducted in 2021 by Saravi et al. [57], the five-year survival of CAD/CAM-produced ceramic multiple unit prostheses was seen at 91.1%. In a systematic review in 2015, all-ceramic prostheses had a lower survival rate than porcelain-fused-to-metal prostheses. Repairing techniques for ceramics include surface preparation of the ceramics and silane treatment in the bonding procedure, which can thus be implemented in further research.

The current review didn’t consider the different products commercially available in the market, i.e. metals and ceramic materials, type of manufacturing method, and powder build-up technique of ceramics. Future reviews can consider these factors to decide which method or material will give the best survival with a cost-effective treatment option for the patient.

## Conclusions

Our study compared the survival rates of two material systems used to fabricate fixed dental prostheses. Failure of either system is often multi-faceted. Failure of either material depends on both the patient and the dentist. For the past ten years, both material systems have been shown to be effective in patients. Within the limitations, the present systematic review found an approximate 99.5% survival rate for PFM tooth-supported prostheses and an approximately 92% survival rate for all-ceramic tooth-supported prostheses after five years of insertion. The most commonly observed complications related to materials were veneer chipping fractures.
